# CytR Is a Global Positive Regulator of Competence, Type VI Secretion, and Chitinases in *Vibrio cholerae*

**DOI:** 10.1371/journal.pone.0138834

**Published:** 2015-09-24

**Authors:** Samit S. Watve, Jacob Thomas, Brian K. Hammer

**Affiliations:** School of Biology, Georgia Institute of Technology, Atlanta, Georgia, United States of America; Ghent University, BELGIUM

## Abstract

The facultative pathogen *Vibrio cholerae* transitions between its human host and aquatic reservoirs where it colonizes chitinous surfaces. Growth on chitin induces expression of chitin utilization genes, genes involved in DNA uptake by natural transformation, and a type VI secretion system that allows contact-dependent killing of neighboring bacteria. We have previously shown that the transcription factor CytR, thought to primarily regulate the pyrimidine nucleoside scavenging response, is required for natural competence in *V*. *cholerae*. Through high-throughput RNA sequencing (RNA-seq), we show that CytR positively regulates the majority of competence genes, the three type VI secretion operons, and the four known or predicted chitinases. We used transcriptional reporters and phenotypic analysis to determine the individual contributions of quorum sensing, which is controlled by the transcription factors HapR and QstR; chitin utilization that is mediated by TfoX; and pyrimidine starvation that is orchestrated by CytR, toward each of these processes. We find that in *V*. *cholerae*, CytR is a global regulator of multiple behaviors affecting fitness and adaptability in the environment.

## Introduction

*Vibrio cholerae* is the causative agent of the diarrheal disease cholera and occupies a range of freshwater and marine environments. The bacterium has been found in association with plants, algae, cyanobacteria, fish, and marine and freshwater invertebrates [1] and its attachment to copepods has been implicated in disease transmission [2]. *Vibrios* and other chitinolytic bacteria degrade the chitinous surfaces of copepods and zooplankton to soluble (GlcNAc)n oligosaccharides that are then imported and utilized as a carbon source [3].

When *V*. *cholerae* associates with chitin, in addition to chitin utilization enzymes, it also produces a DNA uptake apparatus for natural transformation [4]. Components of this apparatus include a pilus that extends into the extracellular environment as well as inner and outer membrane channels that transport DNA molecules into the cytoplasm where it can recombine, allowing horizontal gene transfer [5, 6]. It was recently discovered that in *V*. *cholerae*, growth on chitin induces expression of the Type VI secretion system (T6SS), an apparatus that penetrates and delivers toxic effectors into the cytoplasm of neighboring cells, causing contact-dependent lysis [7]. Lysed cells liberate DNA that can then be used for natural transformation [8].

Genes of the chitin utilization program, natural transformation, and the Type VI secretion system are under the control of a common regulator TfoX, induced by growth on chitin [4]. TfoX is post transcriptionally activated by the TfoR sRNA in response to GlcNAc oligomers liberated from chitinous material [3, 9], but the means by which TfoX activates its downstream targets is poorly understood. A current model suggests that *Haemophilus influenzae* Sxy, a TfoX homolog, may directly activate competence gene promoters by interaction with the cAMP receptor protein (CRP) [10, 11]. However, direct binding of TfoX to its putative target promoters in *V*. *cholerae* has not been demonstrated.

Quorum sensing at high cell density, mediated by the regulator HapR, is also required for natural transformation and Type VI secretion in *V*. *cholerae* in response to secreted autoinducer signals at high density [4, 8, 12, 13]. HapR accumulation down-regulates transcription of the gene for a secreted deoxyribonuclease (*dns*) via direct promoter binding, facilitating DNA uptake by reducing extracellular DNA degradation [14, 15]. HapR also directly activates transcription of the gene coding QstR, a transcriptional regulator that positively controls expression of the periplasmic DNA binding protein *comEA* in the presence of TfoX and the three gene clusters encoding the Type VI secretion system [8, 15]. The mechanism by which QstR activates its target genes is not known, but may require a putative co-factor [11, 15].

We previously identified another regulator, CytR, which positively regulates competence by transcriptional activation of two genes, *comEA* and *pilA*, and upregulates chitin utilization by activation of the chitinase gene *chiA-1* [16]. In *Escherichia coli*, the cytidine repressor CytR negatively regulates a small set of nucleoside scavenging and metabolism genes, including *udp*, *cdd*, *ompK (tsx)*, and *cytR* itself via a CRP-dependent anti-activation mechanism [17]. CRP binding sites in the *udp* promoter of *E*. *coli* allow transcriptional activation by recruitment of RNA polymerase (RNAP) [18, 19]; but specific spacing of two DNA-bound CRP dimers also stabilizes weak CytR-DNA binding interactions that inhibit RNAP recruitment. *V*. *cholerae* CytR represses *udp* transcription in *V*. *cholerae* and in an *E*. *coli cytR* deletion mutant [16, 20]. Thus, CytR in *V*. *cholerae* behaves as a negative regulator of the *udp* nucleoside scavenging gene, as in *E*. *coli*, and also serves as a positive regulator of one chitinase and two competence genes.

Here we show that CytR, like TfoX and HapR, is a global regulator in *V*. *cholerae*. In addition to repressing multiple nucleoside scavenging and metabolism genes, transcriptome analyses demonstrate that CytR also positively regulates the majority of known competence genes, the three known Type VI secretion system (T6SS) gene clusters, and four chitinase genes in *V*. *cholerae*. Distinct regulatory patterns reveal that the specific mechanism of regulation and the participation of each transcription factor differ for each of the three phenotypes studied.

## Results

### CytR is a global regulator in *Vibrio cholerae*

**Fig 1:**
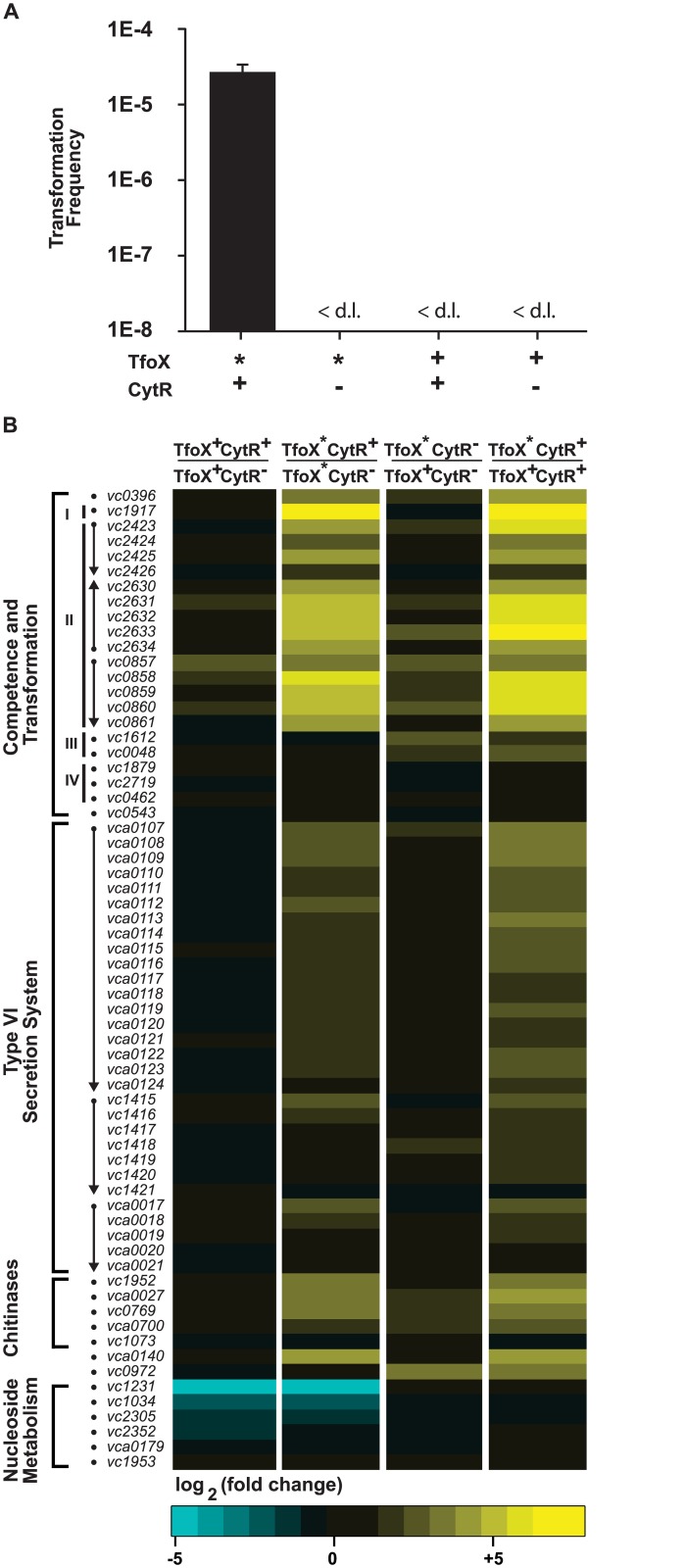
CytR and TfoX co-regulate natural competence, chitinase expression and the type VI secretion system. Panel A: *V*. *cholerae* C6706 is capable of natural transformation in LB medium lacking chitin if *tfoX* is constitutively expressed (TfoX*, bar 1) but not if *tfoX* is under control of its native promoter (TfoX^+^, bars 3 and 4). No transformants were detected in the absence of CytR (CytR^-^, bars 2 and 4). Transformation frequency is expressed as the number of kanamycin resistant cfu mL^-1^ divided by total cfu mL^-1^. The limit of detection (d.l.) is 1 x 10^−8^. Data are shown as mean ± standard deviation from three independent biological replicates. Panel B: Heat map of genes differentially regulated by CytR in the absence (TfoX^+^, column 1) or presence (TfoX*, column 2) of TfoX induction, and genes differentially regulated by TfoX in the absence (CytR^-^, column 3) or presence (CytR^+^, column 4) of a functional *cytR* gene. The majority of known competence genes are positively regulated by both TfoX and CytR and can be classified into four distinct regulatory classes (see text for details). CytR and TfoX positively regulate the three known T6SS gene clusters as well as four chitinase genes. CytR negatively regulates nucleoside uptake and catabolism genes in a TfoX-independent manner.

In *Escherichia coli*, the cytidine repressor CytR negatively regulates a small set of pyrimidine nucleoside scavenging and metabolism genes, including uridine dephosphorylase, *udp* [17]. In *Vibrio cholerae* (El Tor strain C6706), we recently demonstrated that in addition to repressing *udp*, CytR also positively regulates competence genes *comEA* and *pilA*, and the chitinase gene *chiA-1* [16]. We now find that CytR is required for expression of the majority of known competence genes, the three Type VI secretion system (T6SS) clusters, and four known chitinase genes (Fig 1B).

In our prior study, CytR-mediated regulation was measured in a strain with a *luxO* deletion (Δ*luxO*) and a chromosomal *tfoX* allele (tfoX*) under control of a heterologous *ptac* promoter, constitutively expressed due to the lack of a functional LacI repressor in *V*. *cholerae* C6706. The strain is also merodiploid for *hapR* (an additional copy of *hapR* under its native promoter is integrated at the *lacZ* locus) to avoid bypass suppressor mutations in *hapR*, which often occur in lab settings and render C6706 deficient in HapR-dependent quorum sensing [21]. In LB medium this Δ*luxO* Δ*lacZ*:hapR tfoX* strain (EA349) expresses both TfoX and HapR independent of chitin or high cell density, respectively [16]. As a result, EA349 (denoted here as TfoX* CytR^+^) is transformable in LB, whereas its isogenic Δ*luxO* Δ*lacZ*:hapR tfoX* Δ*cytR* mutant (TfoX* CytR^-^) is not (Fig 1A). A Δ*luxO* Δ*lacZ*:*hapR* strain that carries the native *tfoX* allele (denoted here as TfoX^+^ CytR^+^) and an isogenic Δ*luxO* Δ*lacZ*:*hapR* Δ*cytR* strain (TfoX^+^ CytR^-^) are also not transformable with antibiotic-marked linear DNA because the native *tfoX* allele is poorly expressed in the absence of chitin. Thus transformation in LB medium requires the tfoX* allele (TfoX*) and the presence of the native *cytR* allele (CytR^+^).

### Transcriptome analysis

To define the set of genes regulated by CytR in *V*. *cholerae* we performed high throughput RNA sequencing (RNA-seq) on triplicate samples of each of these four strains (TfoX*^/+^ CytR^+/-^) grown to mid-log phase (OD_600_ of 0.5–0.7) in LB medium. In total, 12 RNA samples were sequenced, generating over 216 million 100 bp paired end reads, which were then mapped to the reference genome (El Tor *V*. *cholerae* N16961) and read counts were obtained. Relative abundance of each transcript was determined by applying the RPKM correction to the read counts and pairwise comparisons between different strains were used to calculate fold changes in gene expression [22]. Data were analyzed for genes with transcript abundance differences ≥ 2-fold. Replicate samples showed a high degree of correlation (R>98%).

### CytR negatively regulates nucleoside metabolism in *Vibrio cholerae*

To measure the effect of CytR in the absence of TfoX induction, transcript abundance of the TfoX^+^ CytR^+^ strain (carrying the native *tfoX* allele) was compared to the isogenic TfoX^+^ CytR^-^ mutant. Increased expression of 23 genes and decreased expression of 29 genes was observed in the TfoX^+^ CytR^+^ strain (S1 Dataset). Consistent with prior studies [16, 20], the set of CytR-repressed genes included *udp* (*vc1034*) (Fig 1B column 1 and 2, blue). Negative regulation was also observed for *E*. *coli* homologs of additional nucleoside metabolism genes including: cytidine deaminase, *cdd* (*vc1231*); outer membrane nucleoside transporter, *ompK* (*vc2305*); and one of three genes annotated in *V*. *cholerae* as putative inner membrane nucleoside uptake transporter *nupC*, (*vc2352*) [23]. The *E*. *coli* homolog of *ycdZ*, a putative inner membrane Nup protein [24] was also repressed, while the other two putative *nupC* homologs (*vca0179* and *vc1953*) were not. Gumpenberger and coworkers have recently reported that all three *nupC* homologs are repressed by CytR in *V*. *cholerae* [25]. However, these experiments were performed in minimal media using a transcriptional reporter, which may account for the observed differences. The promoters of these five CytR-repressed genes each have the canonical motif for direct CytR anti-activation (S1 Fig). The remaining four genes experimentally shown to be directly repressed by CytR in *E*. *coli* (*ppiA*, *deoC*, *rpoH* and *nupG*) either lack an obvious *V*. *cholerae* homolog (*nupG* and *ppiA*), or do not appear to be under CytR control in *V*. *cholerae* and lack a typical CytR-binding motif (*deoC* and *rpoH*). These results are consistent with *V*. *cholerae* CytR serving as a negative regulator (anti-activator) of nucleoside scavenging by direct binding.

Based on our prior results that CytR positively regulates *comEA* and *pilA* in conditions where TfoX is induced [16], we hypothesized that additional competence genes may be CytR-controlled in a tfoX* strain, and therefore compared the transcript abundance of different genes in the TfoX* CytR^+^ strain to the isogenic TfoX* CytR^-^ strain. A total of 42 genes showed negative regulation by CytR, including the nucleoside metabolism genes (Fig 1B column 2 blue, and S1 Dataset). However a large number of genes, 84, were also positively regulated (S1 Dataset). In particular, the set of upregulated genes included the transcription factor *qstR*, 15 (of 21) known genes required for natural transformation; 21 genes for the type VI secretion system (T6SS); 4 (of 5) predicted or experimentally validated chitinases; and one chitin utilization gene (Fig 1B column 2 yellow), discussed below.

### CytR positively regulates the majority of known transformation genes

Prior studies have identified 21 genes encoded in 11 loci that are necessary for efficient natural transformation [6, 26]. Under conditions in which *tfoX* was induced, CytR upregulated 15 of these 21 genes by ≥2 fold (Fig 1B column 2, yellow). Consistent with our prior study, *comEA* (*vc1917*) and *pilABCD* (*vc2423-6*) were positively regulated by CytR; in addition to *pilE* (*vc0857*), *vc0858-vc0861*, and *pilMNOPQ* (*vc2634-30*). Interestingly, the HapR-controlled regulatory factor gene *qstR* (*vc0396*) was also upregulated 12-fold by CytR (S1 Dataset). These results confirmed that CytR plays a major role in regulating competence genes in *V*. *cholerae*. In contrast, *pilF* (*vc1612*), *dprA* (*vc0048*), *comEC* (*vc1879*), *comF* (*vc2719*), *pilT* (*vc0462*), and *recA* (*vc0543*) were not under CytR control (Fig 1B, column 3 and 4).

Because the *pilF* homolog, *dprA*, and *comEC* were described as upregulated in response to TfoX induction in El Tor A1552 [6, 26], we also examined our transcriptome data set to determine the effect of tfoX* induction in the C6706 TfoX* CytR^+^ strain as compared to the isogenic TfoX^+^ CytR^+^ strain carrying the native *tfoX* allele in LB medium. We observed a larger number of genes both positively (108 genes) and negatively (25 genes) regulated by TfoX induction, allowing a comparison of our results with those obtained with El Tor strain A1552 [4, 8]. Each CytR-controlled competence gene we identified (in Fig 1B column 2) was also induced by TfoX, consistent with prior studies [4, 8, 26] (S1 Dataset); along with *pilF* and *dprA*, which were upregulated by TfoX but CytR-independent, as in A1552 (Fig 1B column 4 yellow). In C6706 *comEC* was <2-fold induced under all conditions tested (Fig 1, S1 Dataset), in contrast to modest TfoX induction reported in A1552 [15]. We note that our strains are deleted for *luxO* and “locked” at high cell density, unlike the A1552 strains used in previous reports, which may account for these differences.

By comparing the transcript abundance of the TfoX* CytR^-^ strain to the TfoX^+^ CytR^-^ strain, 101 genes were positively controlled, and 47 genes negatively controlled by TfoX induction when *cytR* was absent (Fig 1B column 3, and S1 Dataset). These results are consistent with our prior study showing that despite *tfoX* induction, a Δ*cytR* strain is unable to transcribe *comEA* and *pilA* [16]. Expression of *comF*, *pilT*, and *recA* were under neither CytR nor TfoX control. These results demonstrate that CytR is a critical regulator of the majority of known natural competence genes in *V*. *cholerae*, controlling all but two TfoX-regulated natural transformation genes, *pilF* and *dprA*.

### Differential regulation of competence genes by CytR, TfoX and QstR

**Fig 2:**
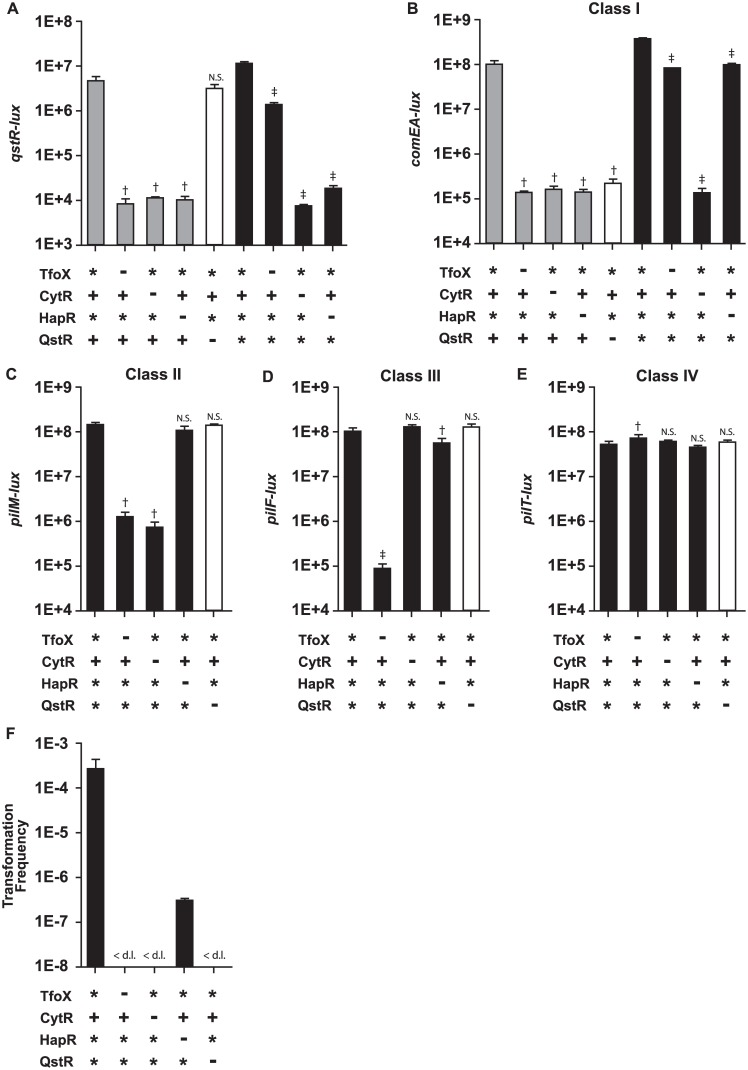
Competence genes are differentially regulated by TfoX, CytR, HapR and QstR. *V*. *cholerae* C6706 derivatives with native alleles of *tfoX*, *cytR* and *qstR* (not constitutively expressed, denoted by +), alleles of *tfoX* or *qstR* made constitutive by replacing the chromosomal native promoter with a *ptac* promoter (indicated by *), or containing in-frame deletions of *tfoX*, *cytR*, *hapR* and *qstR* (-), were analyzed for expression of bioluminescence from plasmid-encoded *lux* transcriptional reporter fusions. Expression profiles are shown for the transcriptional regulator *qstR* (Panel A) and for a member of each regulatory class: class I, *comEA* (Panel B) class II, *pilM* (Panel C) class III, *pilF*, (Panel D), and class IV, *pilT* (Panel E). All strains are deleted for *luxO* and are therefore constitutive for HapR expression (*) when the *hapR* gene is present. Bioluminescence is represented as relative light production per OD_600_ (RLU) and data shown are mean values ± standard deviation from three biological replicates of one representative experiment of three. Data are shown as mean values ± standard deviation. ‡ indicates a p-value < 0.01, † indicates a p-value <0.05. N.S. denotes not significant, calculated using a two-tailed Student’s t-test. In Panels A to E, bars 2–5 are compared to bar 1; in Panels A and B, bars 7–9 are compared to bar 6. Panel F: A TfoX* CytR^+^ HapR* QstR* strain is transformable in LB in the absence of chitin induction, but an isogenic strain carrying a *qstR* deletion was poorly transformable. The *hapR* deletion strain was partially restored for transformation by constitutive expression of QstR (*), but strains deleted for *cytR* or *tfoX* were not restored for competence by the QstR* allele. The limit of detection is 1 x 10^−8^ cfu. mL^-1^ (d.l.).

RNA-seq analyses revealed that both TfoX and CytR positively control the majority of competence genes, (Fig 1), including *qstR*, the transcription factor shown to be directly up-regulated at high cell density by HapR [15]. To confirm and expand upon our RNA-seq observations (Fig 1), we constructed luciferase-based transcriptional fusions to the promoters of the following genes (or operons): *qstR*, *pilM*, *pilE*, *vc0858*, *pilF*, *dprA*, *comF*, *comEC*, and *pilT*. To uncouple native *qstR* expression from HapR, TfoX, and CytR control, a constitutively expressed qstR* allele was also constructed in a manner analogous to the tfoX* allele (see Materials and Methods). Expression of each reporter and of the previously published *comEA-lux* and *pilA-lux* reporters [12, 16] was measured in a *V*. *cholerae* ΔluxO tfoX* (TfoX* CytR^+^ HapR* QstR^+^) strain and in isogenic strains also carrying deletions in *cytR*, *tfoX*, *hapR*, or *qstR* (Fig 2). We find that in agreement with our RNA-seq analysis, competence gene expression falls into four distinct regulatory classes discussed in detail below.

In LB medium, a *V*. *cholerae* ΔluxO tfoX* (TfoX* CytR^+^ HapR* QstR^+^) strain expressed *qstR-lux* robustly while isogenic Δ*tfoX*, Δ*cytR*, and Δ*hapR* mutants were all severely impaired in expression (Fig 2A, grey bars) while the corresponding Δ*qstR* strain was not significantly impaired for expression (Fig 2A, white bar). Interestingly, a constitutive qstR* allele partially restored (about 100-fold increase) expression of *qstR-lux* in the Δ*tfoX* mutant (Fig 2A, compare bars 2 and 7, p < 0.01), suggesting that 1) QstR activates its own transcription and that 2) constitutive expression of QstR also largely bypasses the requirement of TfoX for its activation (Fig 2A). Constitutive QstR expression was not however, able to bypass the requirement for CytR or HapR (Fig 2A, black bars). Thus, expression of QstR is under direct control of HapR, and is controlled by QstR itself, TfoX and CytR, although it remains to be determined whether this is via direct binding.

We have previously reported that the expression of *comEA* depends upon HapR, TfoX and CytR [16]. However, the requirement of HapR for *comEA-lux* expression was bypassed by constitutive expression of QstR (Fig 2B, compare bars 2 and 7, p < 0.01), consistent with our DNA uptake assays (below) and with previous reports [15]. The requirement of TfoX for inducing high levels of *comEA* expression was also bypassed in a qstR* strain (Fig 2B, compare bars 4 and 9, p < 0.01), as also seen by Lo Scrudato and coworkers [11]. By contrast, the requirement of CytR for high levels of *comEA* expression was not restored by constitutive qstR* expression in a Δ*cytR* strain, indicating that *comEA* is positively regulated by QstR and also independently by CytR. Since individual deletions of *tfoX*, *qstR* and *cytR* all result in loss of *comEA* expression, *comEA* is categorized here as a Class I competence gene, requiring all three regulators for expression. Importantly, the qstR* allele bypassed a *hapR* deletion for *comEA* expression, but was unable to restore transformation in either a Δ*tfoX* or Δ*cytR* mutant (Fig 2F).

In contrast to *comEA*, which requires all three transcription factors, maximal expression from the promoters of *pilA*, *pilM*, *vc0857* (*pilE*), and *vc0858* required both CytR and TfoX, consistent with RNA-seq results, but not QstR or HapR (Fig 2C and S1 Dataset) (defined here as Class II competence genes). The requirement of TfoX for expression from these promoters was not bypassed in the strain carrying the qstR* allele (Fig 2C) as it was for *comEA* (Fig 2B). We previously reported that *pilA* expression was dependent on quorum sensing control through HapR [16]. Subsequent independent analyses here show that expression of *pilA* (and *chiA1*, discussed below) does not depend on HapR control, consistent with results reported in *V*. *cholerae* strain A1552 [26] (S2 Fig).

RNA-seq results revealed that expression of *pilF* and *dprA* depend on TfoX as shown by others [4, 27], and similar results were obtained with the *pilF-lux* and *dprA-lux* fusions. These two genes did not require CytR, HapR or QstR for maximal expression (Fig 2D and S2 Fig) and were assigned as Class III competence genes. Consistent with RNA-seq, *pilT-lux* was expressed but not altered in strains deleted for each regulator tested, and the *comEC-lux*, and *comF-lux* reporters were not expressed (Fig 2E and S2 Fig); and were deemed Class IV competence genes because they were not under control of any of the four known regulators based on both RNA-seq and *lux*-based reporter assays.

These data suggest distinct roles for both CytR and TfoX in competence regulation, summarized in the Discussion. TfoX up-regulates *comEA* in a QstR-controlled manner thus dependent on quorum sensing (HapR), while CytR upregulates *comEA* via both QstR-dependent and QstR-independent means. 14 competence genes (*pilE*, *vc0858-0861*, *pilABCD*, and *pilMNOPQ*) are likely regulated by CytR and TfoX in a QstR-independent manner. Also, TfoX up regulates *pilF* and *dprA* in a CytR, QstR and HapR- independent manner. Consistent with this scheme of regulation, we observed that a (TfoX* CytR^+^ HapR* QstR*) strain was highly transformable in LB medium, while no transformants were detected for the corresponding Δ*tfoX*, Δ*cytR*, and Δ*qstR* mutants (Fig 2F). A *hapR*- strain was partially complemented for transformation by QstR* induction similar to results obtained for *V*. *cholerae* strain A1552 [15].

### CytR is required for bacterial killing via the Type VI secretion system

TfoX and QstR positively regulate thirty genes that are encoded in three Type Six Secretion System (T6SS) loci of *V*. *cholerae* (*vc1415-1421*, *vca0017-0021*, and *vca0107-0124*). The T6SS promotes killing of “prey” cells, and the DNA released by the lysed prey can be used for natural transformation [8]. The inner tube of the T6SS apparatus, which also acts as a chaperone for T6SS effectors, requires the membrane ATPase VasK for secretion [28]. RNA-seq analyses revealed that CytR positively regulates all three T6SS operons (Fig 1B, column 2). To characterize the contribution of CytR and the other regulators (TfoX, HapR, and QstR) to T6SS regulation, we constructed luciferase-based transcriptional fusions to the first promoter of the three T6SS loci: *vc1415*, *vca0017*, and *vca0107*. In contrast to competence regulation, the contribution of TfoX and CytR to upregulation of the type VI secretion system occurs primarily through a QstR-dependent mechanism, discussed below.

**Fig 3:**
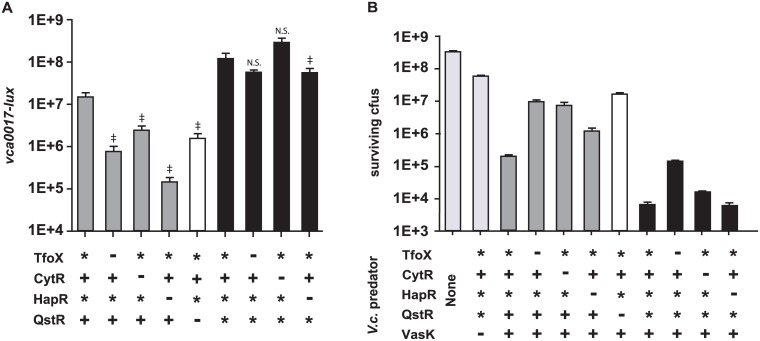
Expression of Type VI secretion system genes and T6SS-mediated killing are positively regulated by CytR, TfoX, HapR, and QstR. *V*. *cholerae* C6706 with indicated alleles of *tfoX*, *cytR*, *hapR*, and *qstR* (+, native;-, deletion; *, constitutively expressed) were analyzed for expression of bioluminescence from a plasmid-encoded *lux* transcriptional reporter fusion to the promoter of first gene of a T6SS auxiliary cluster, *vca0017* (Panel A). Bioluminescence is defined as relative light production per OD_600_ (RLU). All strains are deleted for *luxO* and are therefore constitutive for HapR expression (*) when the *hapR* gene is present. Data shown are mean values ± standard deviation for triplicates from one representative experiment of three performed. ‡ indicates a p-value < 0.01, † indicates a p-value <0.05. N.S. denotes not significant, calculated using a two-tailed Student’s t-test. Bars 2–5 are compared to bar 1 and bars 7–9 are compared to bar 6. Panel B: Chloramphenicol resistant *E*. *coli* prey were incubated with the indicated *V*. *cholerae* predator strains at a ratio of 1:10 on membrane filters to monitor contact-dependent killing. Total surviving prey cfus are represented in each case.

Each T6SS reporter was introduced into the ΔluxO tfoX* strain (TfoX* CytR^+^ HapR* QstR^+^) carrying the native *qstR* allele, an isogenic qstR* strain that expresses QstR constitutively, and derivatives singly deleted for *tfoX*, *cytR*, *hapR*, and *qstR*. Robust expression from the *vca0017-lux* T6SS promoter fusion was observed in the TfoX* CytR^+^ HapR* QstR^+^ strain that expresses QstR from its native promoter (Fig 3A). Expression levels were modestly reduced in the corresponding TfoX^-^ and CytR^-^ strains, supporting the interpretation that CytR, like TfoX, plays a significant role in T6SS gene expression [8]. Expression was lowest in the isogenic HapR^-^ strain, consistent with reports of at least three distinct levels of quorum sensing dependent regulation of T6SS genes: direct binding by quorum regulatory small RNAs [29] and HapR [30], and through QstR [8]. The QstR^-^ strain unable to auto-activate was reduced relative to the parental QstR^+^ strain. Surprisingly, expression of the constitutive qstR* allele bypassed deletion of each of the other four regulators (Fig 3A, black bars). A similar pattern of expression with minor differences was also obtained for luciferase fusions to the other two T6SS promoters (S3 Fig). Thus, heterologous *qstR* expression, uncoupled from its native regulatory role, is sufficient for T6SS gene expression.

Next we investigated whether the type VI secretion system was functional when induced for *tfoX*, *qstR* and *hapR* expression in C6706. Previous reports for the C6706 strain demonstrated that WT C6706 does not express T6SS in LB and is unable to kill *E*. *coli* [8, 31]. Interspecies killing assays between *V*. *cholerae* predator and *E*. *coli* prey performed as described previously [32] revealed robust killing of *E*. *coli* prey by both the TfoX* CytR^+^ HapR* QstR^+^ and TfoX* CytR^+^ HapR* QstR* strains (Fig 3B), but not a *vasK* deletion mutant that is unable to effectively secrete the Hcp subunit of the inner tube of the T6SS [28] or C6706 uninduced for TfoX and HapR (data not shown). Killing was severely impaired in the isogenic TfoX^-^ and CytR^-^ strains consistent with the role of both regulators in T6SS-dependent killing, and the HapR- showed a modest killing defect. By contrast the QstR^-^ strain exhibited no killing, similar to that of the VasK^-^ strain. Consistent with the transcriptional reporter results observed with the qstR* strains (Fig 3A), constitutive qstR* expression bypassed deletions of each of the other regulators for T6SS-mediated killing (Fig 3B, black bars). This mechanism of regulation is distinct from that of transformation. Notably, for T6SS, signal transduction from growth on chitin (via TfoX) and from nucleoside starvation (via CytR) are mediated primarily through QstR; in contrast to TfoX- and CytR-control of transformation, which occurs both through QstR-dependent and QstR-independent pathways (see discussion for details).

### CytR and TfoX co-regulate expression of chitinase genes

**Fig 4:**
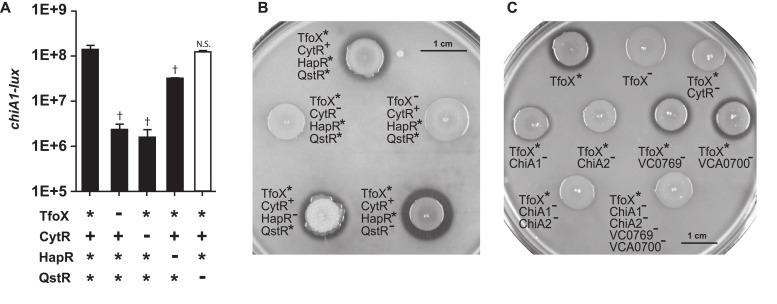
Expression of *V*. *cholerae* chitinases requires TfoX and CytR, but not HapR or QstR. Panel A: *V*. *cholerae* strains with indicated alleles of *tfoX*, *cytR*, *hapR* and *qstR* (+, native;-, deletion; *, constitutively expressed), were analyzed for expression of bioluminescence from a plasmid-encoded *lux* transcriptional reporter fusion to the promoter of the chitinase *chiA1*. All strains are deleted for *luxO* and are therefore constitutive for HapR expression (*) when the *hapR* gene is present. Bioluminescence is defined as relative light production per OD_600_ (RLU). ‡ indicates a p-value < 0.01, † indicates a p-value <0.05. N.S. denotes not significant, calculated using a two-tailed Student’s t-test. Bars 2–5 are compared to bar 1. Panel B and C: Chitin agar plate assays. *V*. *cholerae* strains with indicated alleles of *tfoX*, *cytR*, *hapR*, and *qstR* were assayed for chitinase activity which results in a zone of clearing on LB plates containing 2% colloidal chitin (panel B). Strains constitutive for TfoX (*) and isogenic strains deleted for *cytR*, *tfoX* and the CytR-dependent chitinases *chiA1*, *chiA2*, *vc0769*, *vca0700*, a *chiA1 chiA2* double mutant and a strain deleted for all four chitinases were assayed for the contribution of individual chitinase genes to chitinase activity (panel C).

*V*. *cholerae* has five genes encoding predicted chitinases that may participate in degradation of chitinous material, such as crab and shrimp shells and zooplankton molts [3, 33]. We showed previously that CytR positively regulates expression of a *chiA1-lux* reporter. TfoX was also identified as a critical activator of several predicted or validated chitinase genes (*chiA-1*, *chiA-2*, *vc0769*, and *vca0700*), but not the fifth predicted chitinase *vc1073* [3, 4]. Consistent with these findings, robust up-regulation of each of these four chitinases (but not *vc1073*) was observed in the CytR^+^ TfoX* strain, compared to corresponding strains lacking CytR and TfoX (Fig 1B). To investigate the effect of CytR and TfoX on expression of chitinase genes, we constructed transcriptional luciferase fusions to the promoters of each chitinase and measured expression of these reporters (and of the previously constructed *chiA1-lux* reporter) in a ΔluxO tfoX* qstR* (TfoX* CytR^+^ HapR* QstR*) strain and in isogenic strains carrying deletions in *cytR*, *tfoX*, *hapR* or *qstR*. Maximal expression of each of the four chitinases occurred in the TfoX* CytR^+^ HapR* QstR* strain. Deletions in *cytR* or *tfoX* greatly impaired expression of each reporter, but deletion of *qstR* or *hapR* did not significantly impact chitinase expression (Fig 4A and S4 Fig), suggesting that chitinase expression, like Class II competence gene expression, is controlled by TfoX and CytR, but not HapR or QstR.

To determine the effect of CytR on the ability of *V*. *cholerae* to utilize chitin, we performed a chitin agar plate assay [34]. A TfoX* CytR^+^ HapR* QstR* colony was able to produce a zone of clearing by degradation of colloidal chitin (Fig 4B), while the isogenic TfoX^-^ and CytR^-^ strains did not, indicating that the presence of both CytR and TfoX is necessary for metabolizing chitin. Isogenic HapR^-^ and QstR^-^ strains were able to clear chitin, confirming that quorum sensing is not required for chitinase activity (Fig 4B and S4 Fig). We also tested TfoX* strains singly deleted for each CytR-controlled chitinase (Fig 1B), for the ability to degrade chitin. A TfoX* ChiA-1^-^ mutant produced a decreased zone of clearing while TfoX* ChiA-2^-^ produced a very slight zone of clearing (Fig 4C and S4 Fig). In contrast TfoX* VC0769^-^ and TfoX* VCA0700^-^ strains were not impaired for chitin degradation. Consistent with their predicted role as the dominant extracellular chitinases [3], a ChiA-1^-^, ChiA-2^-^ double mutant produced no zone of clearing, identical to a strain deleted for all four chitinases. (Fig 4C and S4 Fig). Thus, although CytR upregulates four chitinase genes, *chiA1* and *chiA2* appear to be sufficient for degrading colloidal chitin.

## Discussion

**Fig 5:**
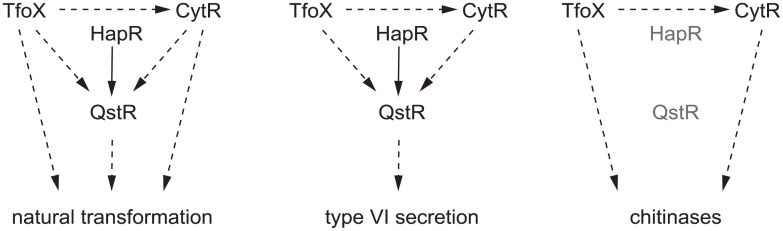
The differential roles of TfoX, CytR, HapR and QstR in natural transformation, Type VI secretion and chitinase expression. Natural transformation requires inputs from four regulators TfoX, CytR, HapR and QstR. Type VI secretion requires inputs from the four regulators above, but QstR overexpression bypasses the need for TfoX, CytR, and HapR. Chitinase expression requires inputs from TfoX, CytR but not from HapR and QstR under the conditions tested.

We have demonstrated that in *V*. *cholerae*, the regulator CytR not only represses genes involved in nucleoside metabolism and transport, but also positively regulates natural transformation, the type VI secretion system, and chitin degradation indicating novel roles for CytR regulation. Although each of these phenotypes requires CytR for expression, the specific mechanism of regulation and the involvement of other transcription factors appear to differ in each case (Fig 5).

Natural transformation requires CytR, chitin-induced TfoX, and quorum sensing-mediated QstR (Fig 5). The majority of competence genes (Class II) require TfoX and CytR for expression but not QstR, while a smaller subset of competence genes are maximally expressed in the presence of all three regulators (Class I) or only TfoX (Class III). The requirement of high cell density conditions (quorum sensing) for regulation of *comEA*, but not for expression of the transformation pilus, suggests a separation in the events of transformation. While CytR and TfoX suffice for the assembly of the transforming pilus and for DNA uptake into the periplasm, subsequent entry into the cytoplasm and potential acquisition of genetic material is contingent upon growth at high cell density. The extracellular DNAse Dns is produced at low cell density, reduces transformation efficiency [14], and is important for utilization of DNA as a phosphate source [35], suggesting that at low cell density, extracellular DNA may be more useful to *V*. *cholerae* in nutrient supply rather than as a source for acquisition of genetic material by horizontal transfer.

In contrast to competence genes, expression of the three T6SS clusters requires TfoX, CytR, HapR and QstR for maximal expression (Fig 3). Quorum sensing control of T6SS expression appears to act multiple levels, via *qrrs*, HapR and QstR [8, 29, 30]. CytR and TfoX are necessary for T6SS expression but their regulatory effects are mediated solely via QstR-upregulation (Fig 5). This suggests a central role for quorum sensing control of killing mediated through T6SS, consistent with a function for T6SS in inter- or intra-species antagonism or in acquiring DNA for natural transformation by lysis of related neighbors [8, 36]

We observed that expression of the four described chitinases in *V*. *cholerae* is dependent on TfoX and CytR and is independent of HapR- and QstR-mediated quorum sensing control, similar to the Class II competence genes (Fig 5). Sun and coworkers observed modest HapR-mediated transcriptional repression of two chitinases, *vca0027* (*chiA-2*) and *vc0769* [37]. However, their experiments were performed with *V*. *cholerae* biofilms on chitin flakes and other levels of signaling may operate under these conditions. These results are consistent with a requirement for chitinase activity both at low cell density during biofilm establishment on chitinous surfaces and at high cell density to ensure a continuous source of carbon.

While expression of TfoX is up-regulated by growth on chitin and QstR expression depends upon both chitin and growth at high cell density, the environmental signals that modulate CytR-dependent regulation are uncertain. TfoX induction increases expression of *cytR* ~2-fold (S1 Dataset and [4]), thus it remains possible that growth on chitin may serve as an activating signal for *cytR* transcription in *V*. *cholerae*. Post-transcriptional regulation results from allosteric binding of cytidine to CytR, which causes de-repression of CytR-repressed targets. In *E*. *coli*, the in vitro binding affinity of CytR for its allosteric ligand cytidine is ~2 μM [38], however de-repression of CytR-repressed targets *in vivo* in both *E*. *coli* and *V*. *cholerae* requires cytidine supplementation at millimolar concentrations unlikely to occur in the environment [16, 39]. It remains possible that the relevant environmental signal may be a modified pyrimidine nucleoside that is more efficiently imported (or less efficiently degraded) and acts as a stable CytR-ligand. Alternatively, CytR may regulate its targets in response to fluctuations in the intracellular nucleotide pools which are known to vary with growth rate and during the stringent response [40].

The mechanism by which CytR up-regulates the phenotypes described here remains to be determined. Rasmussen and coworkers have demonstrated that CytR has the potential to act as a modest transcriptional activator by stabilizing CRP at an artificial weak CRP-binding site [41], but no naturally occurring examples of this type of promoter have been reported. Instead, *in vitro* studies have shown that CytR from *E*. *coli* acts almost exclusively as a repressor (or anti-activator) at a subset of CRP-activated promoters [17]. As a result, we initially proposed that CytR may indirectly act as an activator by repressing another transcriptional factor that directly represses competence genes [16]. However, we have not been able to identify a candidate repressor by genetic screens or chromatin immunoprecipitation followed by high throughput sequencing (chIP-seq) (data not shown). Several genes are differentially regulated at the transcriptional and post-transcriptional level by the relative abundance of their initiating nucleotide [42–44]. Thus it remains possible that CytR may maintain high intracellular UTP and CTP concentrations by repressing pyrimidine catabolism, thereby promoting transcription of genes with pyrimidine initiation nucleotides.

In conclusion, we have demonstrated that CytR, previously thought to function almost exclusively in the nucleoside scavenging response, also regulates genes under chitin and quorum sensing control. Further studies are needed to determine both the relationship between these environmental signals and the molecular mechanism by which this regulation occurs. CytR in *V*. *cholerae* controls multiple behaviors that are important for its fitness and adaptability in the environment.

## Materials and Methods

### Bacterial strains, plasmids, and culture conditions

All *V*.*cholerae* strains were derivatives of a streptomycin resistant C6706 El Tor biotype O1 strain (BH1514), and all *E*.*coli* strains were derivatives of MG1655 and are described in detail (S2 Table). Bacteria were commonly grown at 37°C in Luria broth (LB) under constant shaking, or statically on petri plates containing LB agar, supplemented with ampicillin (100 μg/mL), kanamycin (50 μg/mL), chloramphenicol (10 μg/mL for *V*. *cholerae* and 25 μg/mL for *E*.*coli*), diaminopimelic acid (DAP 50 μg/mL), and streptomycin (5 mg/mL) where appropriate.

### Construction of genetically modified strains of *Vibrio cholerae*

In-frame deletions and promoter-replacement mutants in *V*. *cholerae* were constructed by allelic exchange using pKAS32-based plasmids [45] indicated in S2 Table.

### Recombinant DNA techniques

Standard molecular biology-based methods were utilized for DNA manipulations. DNA modifying enzymes and restriction nucleases (Promega and New England Biolabs), Gibson assembly mix (New England Biolabs), Phusion DNA Polymerase (New England Biolabs), and Taq DNA polymerase (Promega) were used following the manufacturer’s instructions. All modified DNA fragments were tested by colony PCR and verified by Sanger sequencing (Eurofins).

### Transformation assays

Transformation assays in LB medium were performed as described [46]. Briefly, triplicate *V*. *cholerae* cultures grown overnight in LB medium were pelleted using centrifugation and resuspended in fresh LB to an OD_600_ of ~0.1. Diluted cultures were grown until an OD_600_ of ~ 0.3 was reached, and genomic DNA marked with a kanamycin resistance cassette [12] was then added at a final conc. of 1 μg/mL. Cultures were incubated at 30°C for 24 hours and transformants were assayed by plating on LB agar plates with and without kanamycin. Transformation frequency was defined as Kan^R^ cfu mL^−1^/total cfu mL^−1^.

### RNA-Sequencing

Total RNA from 3 independent cultures of four *V*. *cholerae* strains grown to exponential phase (OD_600_ 0.5–0.7) in LB medium at 37°C was extracted using mirVana miRNA isolation kit (Ambion). DNAse treatment for removal of genomic DNA was performed using TURBO DNA free kit (Ambion). Detection of contaminating genomic DNA was carried out by performing PCR amplification with primers specific for 16S rRNA loci, and DNAse treatment was repeated until no PCR products were detectable. DNA-free total RNA samples were purified using RNEasy Minelute kit (Qiagen). All kits were used as per manufacturers’ instructions unless described otherwise. Further processing of the samples was conducted by Eurofins (Louisville, USA) using a standardized Illumina RNA Sequencing pipeline. Briefly, RNA sample quality was determined using an Agilent 2100 Bioanalyzer and Qubit, before ribodepletion using a Ribo-Zero Magnetic kit (Epicentre) for Gram-negative bacteria. Sequencing was performed using a HiSeq2000 sequencer (Illumina) and 100bp paired end reads were obtained.

Reads were mapped to chromosomes I and II of *V*. *cholerae* N16961 (European nucleotide archive accession numbers AE003852.1and AE003853.1) using Bowtie2 [47]. Mapped reads were visualized using Seqmonk v2.8 (Babraham Bioinformatics) and read counts obtained using Seqmonk’s RNA-seq Quantitation pipeline. Statistical analysis for differentially expressed genes was performed using the DESeq package [48] and genes with > 2 fold change and p-value <0.05 were analyzed. For the RNA-seq statistics and entire list of differentially expressed genes, see S1 Table and S1 Dataset. Heat maps were generated using R statistical package (v 3.0.2). [49]

### Bioluminescence Assays

*V*. *cholerae* strains carrying lux-based reporter plasmids were grown on LB agar plates containing chloramphenicol at 37°C overnight. Cells were resuspended in LB medium containing chloramphenicol to an initial OD_600_ of 0.01 and incubated with shaking at 37°C until an OD_600_ of 0.8–1.0 was reached. Bioluminescence and absorbance were quantified as described previously [50]. Bioluminescence was measured using a Wallac model 1409 liquid scintillation counter as described previously [51] and optical density of each culture was measured with a spectrophotometer. Relative Light Units (RLU) are defined as counts min^−1^ mL^−1^/OD_600_. Single-time-point experiments were performed in triplicate.

### T6SS killing assay

The T6SS killing assay was modified from previously described methods [32]. *V*. *cholerae* and *E*. *coli* strains grown overnight on LB plates at 37°C were resuspended in LB medium to an OD_600_ of 0.01 and incubated with shaking at 30°C until they reached an OD_600_ of 1.0. Predator and prey strains were mixed at a ratio of 10:1 and 50 μL of each suspension was spotted onto sterile Whatman cellulose gridded filters (GE Healthcare) placed on LB plates. After incubation at 37°C for 3 hours, filters were removed and washed with 5 mL LB medium to recover cells. Dilutions of the cell suspension were plated on LB agar supplemented with chloramphenicol to determine counts of surviving prey.

### Chitinase plate assay

Colloidal chitin was prepared from practical grade chitin (Sigma) derived from shrimp shells as previously described [34, 52]. Colloidal chitin plates were made by mixing 2% w/v colloidal chitin with LB medium buffered to pH 7.0 with 0.1M phosphate buffer. Strains were incubated overnight at 37°C in LB broth, diluted to an OD_600_ of 1.0 and 10 μL of each suspension was stabbed into the chitin agar. The plates were incubated at 37°C for 72 hours and the zone of chitin clearing for each colony was recorded.

## Supporting Information

S1 Dataset Differential regulation of genes by CytR and by TfoX in *V*. *cholerae*. Raw counts of the transcripts obtained from coding regions were calculated and pairwise comparisons were made to calculate relative fold-change as described in the text. A fold change >2 and a p value <0.05 denotes positive regulation while a fold change <0.5 indicates negative regulation. Data in each tab of the spreadsheet refers to individual columns described in Fig 1B of the main text.
(XLSX)

S1 Fig Predicted CRP binding site pairs of nucleoside catabolism and transport genes anti-activated by CytR. Putative CytR binding sites were determined by identifying two CRP binding sites (highlighted in grey) in the -200 to +100 region (with respect to translational start site) of each target gene separated by a spacing of 49–53 nucleotides. CRP sites were determined by FIMO (C.E. Grant, T.L Bailey and W.S. Noble, Bioinformatics 27:1017–18, 2011) TGTGA-N6-TCACA (p < 0.01).
(EPS)

S2 Fig Differential regulation of competence genes by TfoX, CytR, HapR, and QstR. *V*. *cholerae* strains in which the regulators *tfoX*, *qstR*, *cytR* and *hapR* were present (+), deleted (-) or constitutively induced (*) were analyzed for bioluminescence from the following plasmid-encoded transcriptional reporters: *pilA-lux* (Panel A), *vc0857-lux* (Panel B), *vc0858-lux* (Panel C), *dprA-lux* (Panel D), *comEC-lux* (Panel E), and *comF-lux* (Panel F). Bioluminescence is represented as relative light production per OD_600_ (RLU). Data shown are mean values ± standard deviation for biological triplicates. ‡ indicates a p-value < 0.01, † indicates a p-value <0.05. N.S. denotes not significant, calculated using a two-tailed Student’s t-test. Bars 2–5 are compared to bar 1.
(EPS)

S3 Fig Differential regulation of T6SS clusters by TfoX, CytR, HapR, and QstR. *V*. *cholerae* strains carrying the indicated alleles of the regulators *tfoX*, *qstR*, *cytR* and *hapR* (+, native;-, deletion; *, constitutively expressed) were analyzed for bioluminescence from the plasmid-encoded transcriptional reporters *vc1415-lux* (Panel A) and *vca0107-lux* (Panel B). Bioluminescence is represented as relative light production per OD_600_ (RLU). Data shown are mean values ± standard deviation for biological triplicates. ‡ indicates a p-value < 0.01, † indicates a p-value <0.05. N.S. denotes not significant, calculated using a two-tailed Student’s t-test. Bars 2–5 are compared to bar 1 and bars 7–9 are compared to bar 6.
(EPS)

S4 Fig Differential regulation of chitinases by TfoX, CytR, HapR, and QstR. *V*. *cholerae* strains in which the regulators *tfoX*, *qstR*, *cytR* and *hapR* were present (+), deleted (-), or constitutively induced (*) were analyzed for bioluminescence from the following plasmid-encoded transcriptional fusions to chitinase promoters: *chiA2-lux* (Panel A), *vca0700-lux* (Panel B), *vc0769-lux* (Panel C) and *vc1073-lux* (Panel D). Bioluminescence is represented as relative light production per OD_600_ (RLU). Data shown are mean values ± standard deviation for biological triplicates. ‡ indicates a p-value < 0.01, † indicates a p-value <0.05. N.S. denotes not significant, calculated using a two-tailed Student’s t-test. Bars 2–5 are compared to bar 1. (Panel E) Chitin agar plate assay. Indicated strains were assayed for the ability to degrade colloidal chitin and produce a visible zone of clearing. Measurements show representative values for mean colony diameter and zone of clearing as well as standard deviation obtained from 9 biological replicates for each strain tested.
(EPS)

S1 Table Summary statistics of RNA-seq. 12 Multiplexed cDNA libraries were derived from DNA-depleted *Vibrio cholerae* total RNA and sequenced to give 100 bp paired end reads as described in Materials and Methods. >98% of the 216 million reads obtained mapped onto the reference genome of *Vibrio cholerae* N16961 (J. F. Heidelberg, J. A. Eisen, W. C. Nelson, R A. Clayton, et al. Nature 406(6795): 477–483.) obtained from EBI.
(DOCX)

S2 Table List of strains and plasmids used in this study. (DOCX)
