# Supplementary material for: CytR Is a Global Positive Regulator of Competence, Type VI Secretion, and Chitinases in Vibrio cholerae
Source: PLoS One. 2015 Sep 24;10(9):e0138834. doi: 10.1371/journal.pone.0138834 (PMC4581735; doi:10.1371/journal.pone.0138834)
Supplement: S1 Table — 12 Multiplexed cDNA libraries were derived from DNA-depleted Vibrio cholerae total RNA and sequenced to give 100 bp paired end reads as described in Materials and Methods. >98% of the 216 million reads obtained mapped onto the reference genome of Vibrio cholerae N16961 (J. F. Heidelberg, J. A. Eisen, W. C. Nelson, R A. Clayton, et al. Nature 406(6795): 477–483.) obtained from EBI. (DOCX) [file pone.0138834.s006.docx]

**Table S1** Summary statistics of RNA-seq.

| **Sample Name** | **Genotype** | **Index** | **Yield (GB)** | **# Reads (Millions)** | **% Reads Q >=30** | **Mean Q Score** | **% Reads aligned to reference genome** |
| --- | --- | --- | --- | --- | --- | --- | --- |
| A | Δ*luxO*, *tfoX**, Δ*cytR*, Δ*lacZ*:*hapR* | ATCACG | 1.89 | 18.89 | 86.7 | 34.12 | 99.18 |
| B | Δ*luxO*, *tfoX**, Δ*cytR*, Δ*lacZ*:*hapR* | CGATGT | 2.11 | 21.09 | 87.36 | 34.3 | 98.79 |
| C | Δ*luxO*, *tfoX**, Δ*cytR*, Δ*lacZ*:*hapR* | TTAGGC | 1.92 | 19.20 | 86.6 | 34.08 | 98.39 |
| D | Δ*luxO*, *tfoX**, Δ*lacZ*:*hapR* | TGACCA | 2.18 | 21.79 | 86.77 | 34.12 | 98.72 |
| E | Δ*luxO*, *tfoX**, Δ*lacZ*:*hapR* | ACAGTG | 1.76 | 17.56 | 86.87 | 34.15 | 98.6 |
| F | Δ*luxO*, *tfoX**, Δ*lacZ*:*hapR* | GCCAAT | 1.9 | 18.98 | 86.91 | 34.17 | 98.44 |
| G | Δ*luxO*, Δ*cytR*, Δ*lacZ*:*hapR* | CAGATC | 1.8 | 18.02 | 87.43 | 34.31 | 99.41 |
| S | Δ*luxO*, Δ*cytR*, Δ*lacZ*:*hapR* | ACTTGA | 1.81 | 18.05 | 87.46 | 34.32 | 99.39 |
| I | Δ*luxO*, Δ*cytR*, Δ*lacZ*:*hapR* | GATCAG | 1.45 | 14.46 | 87.76 | 34.42 | 99.44 |
| J | Δ*luxO*, Δ*lacZ*:*hapR* | TAGCTT | 1.55 | 15.53 | 87.5 | 34.33 | 99.39 |
| K | Δ*luxO*, Δ*lacZ*:*hapR* | GGCTAC | 1.74 | 17.43 | 87.64 | 34.38 | 99.41 |
| L | Δ*luxO*, Δ*lacZ*:*hapR* | CTTGTA | 1.51 | 15.14 | 87.82 | 34.42 | 99.3 |
|  |  | Total | 21.62 | 216.14 |  |  |  |
