# Supplementary material for: CytR Is a Global Positive Regulator of Competence, Type VI Secretion, and Chitinases in Vibrio cholerae
Source: PLoS One. 2015 Sep 24;10(9):e0138834. doi: 10.1371/journal.pone.0138834 (PMC4581735; doi:10.1371/journal.pone.0138834)
Supplement: S2 Table — (DOCX) [file pone.0138834.s007.docx]

| **Strains** | **Genotype or Description** | **Reference** |
| --- | --- | --- |
| SW051 | *ΔluxO ptac-tfoX ΔcytR Δlacz:HapR* | This study |
| EA349 | *ΔluxO ptac-tfoX Δlacz:HapR* | E.S. Antonova, E. E. Bernardy and B. K. Hammer, Mol. Microbiol. **86**:1215-31, 2012 |
| EA432 | *ΔluxO ΔcytR Δlacz:HapR* | This study |
| EA372 | *ΔluxO Δlacz:HapR* | This study |
| EA281 | *∆luxO ptac-tfoX* | E.S. Antonova, E. E. Bernardy and B. K. Hammer, Mol. Microbiol. **86**:1215-31, 2012 |
| JT100 | *∆luxO ptac-tfoX ptac-qstR* | This study |
| JT708 | *∆luxO ∆tfoX* | This study |
| JT710 | *∆luxO ∆tfoX ptac-qstR* | This study |
| EA637 | *∆luxO ptac-tfoX ∆cytR* | E.S. Antonova, E. E. Bernardy and B. K. Hammer, Mol. Microbiol. **86**:1215-31, 2012 |
| JT98 | *∆luxO ptac-tfoX ∆cytR ptac-qstR* | This study |
| JT128 | *∆luxO ∆hapR ptac-tfoX* | This study |
| JT127 | *∆luxO ∆hapR ptac-tfoX ptac-qstR* | This study |
| JT129 | *∆luxO ∆qstR ptac-tfoX* | This study |
| EA90 | *lacZ:KanR* | E.S. Antonova and B.K. Hammer, FEMS Microbiol. Lett. **322**:68-76, 2011 |
| EA305 | *ptac-tfoX* | This study |
| EA355 | *ΔtfoX* | This study |
| EA431 | *ptac-tfoX ΔcytR* | This study |
| SW323 | *ptac-tfoX-Chr ΔchiA1* | This study |
| SW336 | *ptac-tfoX-Chr ΔchiA2* | This study |
| SW352 | *ptac-tfoX Δvc0769* | This study |
| JT603 | *ptac-tfoX ΔvcA0700* | This study |
| SW358 | *ptac-tfoX-Chr ΔchiA1 ΔchiA2* | This study |
| SW408 | *ptac-tfoX-Chr ΔchiA1 ΔchiA2 ∆vc0769* | This study |
| SW410 | *ptac-tfoX-Chr ΔchiA1 ΔchiA2 ∆vc0769 ∆vca0070* | This study |
| NRD204 | *E. coli* MG1655 *∆araBAD::cat* | N. R. De Lay and J. E. Cronan, J. Biol. Chem. **282.28**: 20319-28, 2007 |
| C6706str2 | C6706 El Tor biotype O1 | K. H. Thelin and R. K. Taylor, Infect. Immun. **64(7):** 2853-2856, 1996 |
| JT652 | *∆luxO ptac-tfoX ptac-qstR ∆vasK* | This study |

**Table S2** List of strains and plasmids used in this study.

| **Plasmids** | **Features** | **Reference** |
| --- | --- | --- |
| pBBRlux | Cloning vector, CmR | D. H. Lenz, K. C. Mok, B. N. Lilley, R. V. Kulkarni, N. S. Wingreen, and B. L. Bassler, Cell **118(1):**69-82, 2004 |
| pEA209 | *comEA–lux*, CmR | [E.S. Antonova and B.K. Hammer, FEMS Microbiol. Lett. **322**:68-76, 2011](http://onlinelibrary.wiley.com/enhanced/doi/10.1111/mmi.12054/#mmi12054-bib-0001) |
| pEA493 | *pilA–lux*, CmR | E.S. Antonova, E. E. Bernardy and B. K. Hammer, Mol. Microbiol. **86**:1215-31, 2012 |
| pJT315 | *pilF–lux*, CmR | This study |
| pJT313 | *pilM–lux*, CmR | This study |
| pJT314 | *pilT–lux*, CmR | This study |
| pSW149 | *qstR-lux*, CmR | This study |
| pJT712 | *vc0857–lux*, CmR | This study |
| pJT260 | *vc0858–lux*, CmR | This study |
| pJT261 | *comEC–lux*, CmR | This study |
| pJT353 | *comF–lux*, CmR | This study |
| pJT711 | *vc0047-lux*, CmR (*dprA* operon) | This study |
| pEA495 | *chiA1–lux*, CmR | E.S. Antonova, E. E. Bernardy and B. K. Hammer, Mol. Microbiol. **86**:1215-31, 2012 |
| JT579 | *chiA2–lux*, CmR | This study |
| JT580 | *vca0700–lux*, CmR | This study |
| JT581 | *vc0769–lux*, CmR | This study |
| JT582 | *vc1073–lux*, CmR | This study |
| pJT653 | *vca0017-lux*, CmR | This study |
| pJT654 | *vc1415-lux*, CmR | This study |
| pJT660 | *vca0107-lux*, CmR | This study |
